# Effect of Ba Content on the Activity of La_1‐*x*_Ba_*x*_MnO_3_ Towards the Oxygen Reduction Reaction

**DOI:** 10.1002/celc.201800052

**Published:** 2018-04-06

**Authors:** Gael. P. A. Gobaille‐Shaw, Veronica Celorrio, Laura Calvillo, Louis J. Morris, Gaetano Granozzi, David. J. Fermín

**Affiliations:** ^1^ School of Chemistry University of Bristol Cantocks Close Bristol BS8 1TS UK; ^2^ EPSRC Centre for Doctoral Training in Catalysis School of Chemistry Cardiff University Main Building Park Place Cardiff CF10 3AT UK; ^3^ Dipartimento di Scienze Chimiche Università di Padova Via Marzolo 1 35131 Padova Italy; ^4^ UK Catalysis Hub, Research Complex at Harwell RAL, Oxford, OX11 0FA, UK and Kathleen Lonsdale Building Department of Chemistry University College London Gordon Street London WC1H 0AJ UK

**Keywords:** Electrocatalysis, kinetics, oxygen reduction reaction, La_1-*x*_Ba_*x*_MnO_3_, perovskite nanoparticles

## Abstract

The electrocatalytic activity of La_1‐*x*_Ba_*x*_MnO_3_ nanoparticles towards the oxygen reduction reaction (ORR) is investigated as a function of the A‐site composition. Phase‐pure oxide nanoparticles with a diameter in the range of 40 to 70 nm were prepared by using an ionic liquid route and deposited onto mesoporous carbon films. The structure and surface composition of the nanoparticles are probed by XRD, TEM, EDX, and XPS. Electrochemical studies carried out under alkaline conditions show a strong correlation between the activity of La_1‐*x*_Ba_*x*_MnO_3_ and the effective number of reducible Mn sites at the catalysts layer. Our analysis demonstrates that, beyond controlling particle size and surface elemental segregation, understanding and controlling Mn coordination at the first atomic layer is crucial for increasing the performance of these materials.

## 1 Introduction

To accelerate the mass utilisation of advanced energy technologies such as fuel cells and metal‐air batteries, it is crucial to address limiting factors associated with the dynamic of the oxygen reduction (ORR) and evolution reactions.^1^ The development of non‐Pt based electrocatalysts is one of the major technological challenges in the field, with transition metal oxides such as perovskites ABO_3_ being considered as promising candidates under alkaline conditions.^2^,^3^ Establishing structure‐activity relationships in this class of materials have mainly focused on the bulk “ground‐state” descriptors such as orbital occupancy, M−O hybridisation, strain and so forth.^4^, ^5^, ^6^ Lee et al. estimated the formation energy of adsorbed OH intermediate under two different configuration employing DFT at LaBO_3_ (001) (B=Co, Cr, Fe, Ni and Mn), concluding that Co provides the most appropriate configuration.^8^ However, experimental studies by Celorrio *et al*. established that the activity of LaMnO_3_ nanoparticles in carbon supports is orders of magnitude higher than the other transition metal oxide in the series.^9^ Celorrio et al. have also shown that the onset potential for ORR overlaps with the reduction of Mn sites at the surface of the nanostructures.^9^, ^10^, ^11^ The link between changes in oxidation states of Mn sites and ORR activity has been considered in the context of MnO_*x*_ catalysts^2^ and recently confirmed by in‐situ XAS studies.^6^

A review by Stoerzinger et al. illustrates the wide range of activity reported by Mn‐based oxides, putting into perspective the lack of understanding of the basic principle guiding the reactivity of these materials in comparison to metal catalysts.^5^ For instance, the role of the A‐site is yet to be fully rationalised in manganite perovskites. Celorrio et al. investigated the La replacement by Ca, assessing the effect on the Mn oxidation state, bulk structure and surface composition.^10^ Through a detailed analysis of the electrochemical responses of Mn surface sites, it was proposed that Mn site with an effective oxidation state of 2.8 provided the highest intrinsic activity.^10^ Replacing La^3+^ by cations such as Ba^2+^ can induced even stronger effect in the structure of the manganite perovskite, considering the significantly difference in ionic radius. Indeed, Lee et al. found that Ba substitution resulted in profound changes in the reducibility and availability of surface manganese as evidenced by temperature‐programmed reduction (TPR) and desorption (TPD), which also manifest itself in increased catalytic activity towards CO_2_ reforming of CH_4_.^13^ In this study, phase pure La_1‐*x*_Ba_*x*_MnO_3_ nanoparticles with bulk compositions of x=0, 0.15, 0.30 and 1 are synthesised and characterised by XRD, TEM and EDX. The surface composition of the particles was investigated by XPS, while the effective number density of reducible Mn sites was estimated by cyclic voltammetry. The activity of the oxide nanoparticles supported on carbon electrodes towards the ORR was assessed by rotating ring‐disk electrode under alkaline conditions. For the first time, we show that the kinetically limited current is proportional to the square of the number density of reducible Mn sites. The implications of this observation with respect to the ORR mechanism are briefly discussed.

## **Experimental Section**

### Synthesis of La_1‐*x*_Ba_*x*_MnO_3_ Oxide Nanoparticles

La_1‐*x*_Ba_*x*_MnO_3_ nanoparticles were synthesised by a highly versatile ionic‐liquid based method which have generated phase pure lanthanides,^9^ manganites,^10^,^11^ cobaltites,^12^ ferrites,^14^,^15^ and oxyhalides.^16^ Briefly, EDTA (1 : 1 metal nitrate/EDTA) was dissolved in 1‐ethyl‐3‐methylimidazolium acetate (1 mL) at 80 °C. La(NO_3_)_3_.6H_2_ O (0.5 mL, 0.1 M) and Mn(NO_3_)_2_.4H_2_O (0.5 mL, 0.1 M) were then added to the ionic liquid and heated under stirring for 2–3 hrs to dehydrate the precursors. After this period microcrystalline cellulose (100 mg, 10 wt%) was added and allowed to stir at 80 °C for 10 minutes, resulting in a homogenous mixture. The resultant mixture was calcined immediately after in an alumina crucible with a ramp rate of 5 °C per minute. All samples were initially heated to 700 °C with a dwell time of 2 h. La_1‐*x*_Ba_*x*_MnO_3_ required a secondary annealing process of 5 h of 850 °C and 1100 °C for *x*=0.15 and 0.3 to achieve phase purity, whereas BaMnO_3_ was synthesised at 950 °C. The requirement of higher annealing temperatures to incorporate increasing amount of Ba has been previously reported.^17^ The difference in crystallisation temperature has shown to be related to the strain in the crystal lattice induced by the difference in ionic radii between La and Ba.^18^ The use of EDTA, although not essential, ensures that no precipitation occurs during the dehydration at the early stages of the calcination process thus inhibiting formation of secondary oxide phases.

### Characterisation and Electrochemical Measurements

X‐ray diffraction (XRD) of the materials was performed with a Bruker AXS D8 Advance diffractometer equipped with a CuKα radiation source. The structures were also investigated using scanning (JEOL SEM 5600 LV) and transmission electron microscopy (JEOL JEM‐1200). Samples for TEM were produced by placing 1 μL drops of the oxide particles dispersed in ethanol on a 3 mm carbon‐coated copper grid. Mean particle diameters were estimated from at least 100 nanoparticles per sample. Photoemission data was obtained in a custom designed UHV system equipped with an EA 125 Omicron electron analyser with five channeltrons, working at a base pressure of 10^−10^ mbar. Core level photoemission spectra (C 1s, O 1s, Mn 2p, Ba 3d and La 3d regions) were collected in normal emission at room temperature with a non‐monochromatised Al K_α_ X‐ray source (1253.6 eV) and using 0.1 eV steps, 0.5 s collection time and 20 eV pass energy. The binding energies (BE) were referenced to the C 1s peak at 284.6 eV. The surface composition of the samples was obtained from the Ba 4d, Mn 3p and La 4d peak regions taking into account the corresponding sensitivity factors.

The EXAFS measurements were carried out at room temperature at the SAMBA beamline of Soleil Synchrotron (Saclay, France). The beamline is equipped with a sagittally focusing Si 220 monochromator and two Pd‐coated collimating/focusing mirrors to remove higher harmonics. Calibration of the monochromator was carried out using a Mn foil. Samples were pelletised as disks of 10 mm diameter with 1 mm thickness using cellulose powder as a binder, and XAFS spectra were recorded in transmission mode at the Mn K‐edge (6539 eV). A total of three spectra were averaged for each sample. The spectra were aligned using the Mn foil response. The data was analysed using the Athena software.^7^

Electrochemical measurements were carried out using a rotating ring‐disk electrode (glassy carbon disk with geometric surface area of 0.13 cm^2^) operated with an ALS rotation controller and an Ivium‐CompactStat bipotentiostat. The collection efficiency of the RRDE was determined to be 0.42. All experiments were measured using a Hg/HgO reference electrode (0.1 M KOH) with a graphite counter electrode. Electrodes were prepared via a two‐step drop‐casting procedure leading to a controlled loading 250 μg oxide cm^−2^, 50 μg Vulcan cm^−2^ and 50 μg Nafion cm^−2^.^9^, ^10^, ^11^,^19^,^20^ First, the Vulcan/Nafion aqueous suspension is dropped at the electrode surface followed by a volumetric drop of suspended metal oxide nanoparticles and subsequently dried in air.

## 2 Results and Discussion

**Figure 1:**
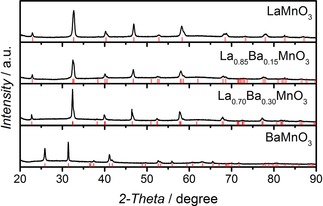
XRD patterns of the different La_1‐*x*_Ba_*x*_MnO_3_ nanoparticles. The red bars correspond to the positions of the allowed Bragg reflections for the main phase. Representative TEM images (Figure S1), particle size distributions (Figure S2), bulk elemental composition (Table S1), and specific surface area values (Table S2) are provided in the Supporting Information.

Figure 1 displays the XRD of the as‐prepared La_1‐*x*_Ba_*x*_MnO_3_ oxide nanoparticles, revealing a high degree of phase purity based on their close match with the allowed Bragg reflections for the main phase (red bars). LaMnO_3_ was indexed to a cubic (*Pm3m*) phase, whereas the mixed composition samples were indexed to the rhombohedral space group (*R‐3cH*) in a hexagonal setting which, is consistent with previous findings by Chakraborty et al.^17^ BaMnO_3_ was indexed to the *P*6_3_ */mmc* hexagonal space group. Quantitative elemental analysis by SEM‐EDX indicates that the bulk elemental stoichiometry is close to the elemental composition of the precursor solution (see Table S1).

TEM images were used to calculate mean particle sizes; representative images are shown in Figure S1 and their particle size histograms can be found in Figure S2. The specific surface areas of the particles (SSA) reported in Table S2 are calculated from the mean particle diameter measured by TEM. The data shows a systematic increase in particle size with increasing Ba content from 33.8±1.6 to 73.5±3.5 nm. This trend leads to a decrease in the SSA from 25.91±1.3 m^2^ g^−1^ (LaMnO_3_) to 13.9±0.7 m^2^ g^−1^ (BaMnO_3_).

**Figure 2:**
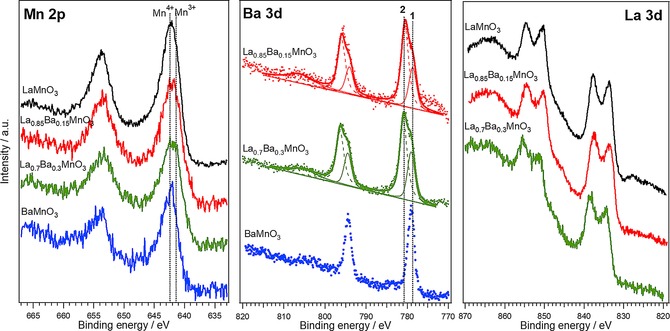
Mn 2p, Ba 3d and La 3d photoemission spectra taken in normal emission using non‐monochromatised Al Kα X‐ray source. The Ba 3d line involves two components associated with the Ba in the perovskite lattice (1) and as Ba oxide/hydroxide (2).

Figure 2 shows the XPS spectra acquired in the Mn 2*p*, Ba 3*d* and La 3*d* regions. The surface composition of the La_1‐*x*_Ba_*x*_MnO_3_ nanoparticles shown in Table S3 has been estimated from the area of the Mn 3*p*, Ba 4*d* and La 4*d* peaks and their corresponding sensitivity factors. LaMnO_3_ and BaMnO_3_ show A/B ratios at the surface not far from the expected bulk ones. On the other hand, there is significant A‐site atomic surface enrichment in the case of the mixed A‐site samples. This is a clear evidence of a surface segregation of La and Ba forming their corresponding oxides/hydroxides which could be rationalised in terms of the higher temperatures required for generating mixed A‐site samples. XPS data also show a significantly different La/Ba ratio at the surface with respect to the bulk one in the case of La_0.70_Ba_0.3_MnO_3_. This could be a manifestation of the strain relaxation process associated with the bulkier Ba^++^ cations which can facilitate the surface segregation.

Figure 2 shows very little dependence of the Mn 2p_5/2_ binding energy (BE) as a function of the La_1‐*x*_Ba_*x*_MnO_3_ composition. The mean oxidation state of Mn sites is expected to be +3 in LaMnO_3_, +4 in BaMnO_3_ and within +3 and +4 in La_1‐*x*_Ba_*x*_MnO_3_ samples. This is substantiated by the X‐ray absorption near‐edge spectra (XANES) at the Mn K‐edge data reported in Figure S3: they clearly show a higher energy for the characteristic pre‐edge feature in the case of BaMnO_3_ with respect to LaMnO_3_. Comparing the pre‐edge position with standard Mn‐oxides, following protocols reported elsewhere,^10^ the bulk Mn oxidation state in LaMnO_3_ and BaMnO_3_ is very close to +3 and +4, respectively. However, the close XPS Mn 2p_5/2_ binding energy (BE) of Mn^3+^ (641.9 eV) and Mn^4+^ (642.2 eV) makes very challenging to quantitatively establish the effective Mn oxidation state at the surface (dotted lines in Figure 2).^21^ The described segregation of La and Ba observed in mixed La/Ba samples (Table S3), with the consequent formation of surface oxides/hydroxides, appears to promote the formation of Mn^4+^ sites at the surface of the nanoparticles. As discussed further below, electrochemical responses appear very sensitive to the coordination of the surface Mn sites.

Figure 2 also shows the characteristic double splitting of the La 3*d* photoemission lines due to the interaction between an electron from the oxygen valence band and the empty La 4*f* level. The La 3*d*_5/2_ BE is located at 834.4 eV, corresponding to La^3+^ compounds.^22^ The BaMnO_3_ sample shows a narrow Ba 3*d*_5/2_ peak centred at 779.0 eV, which can be attributed to the Ba in the perovskite lattice. However, the Ba 3*d*_5/2_ line in La_1‐*x*_Ba_*x*_MnO_3_ samples contains two components, the first one at 779.0 eV associated with Ba in the perovskite lattice, and a second at 780.6 eV attributed to the surface oxide/hydroxide.^23^ This second component has also been linked to the formation of BaCO_3_ on the surface; however, the absence of the corresponding carbonate component in the C 1s line (not shown) does not support this assignment.

**Figure 3:**
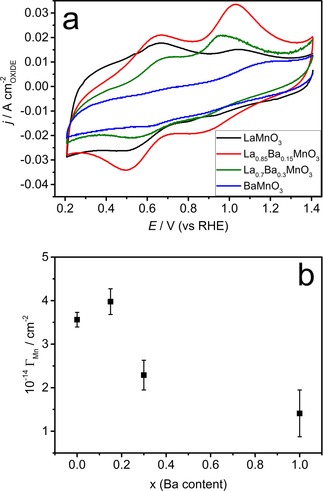
Cyclic voltammograms for BaMnO_3_, La_0.7_Ba_0.3_MnO_3_, La_0.85_Ba_0.15_MnO_3_ and LaMnO_3_ nanoparticles supported at a mesoporous carbon electrode in Ar‐saturated 0.1 M KOH solution at 0.010 V s^−1^ (a). Dependence of the effective number density of reducible Mn site (*Γ*_Mn_) on the Ba bulk content (b).

Figure 3a compares the cyclic voltammograms of the various La_x_Ba_1‐x_MnO_3_ nanoparticles in Ar‐saturated 0.1 M KOH solution at 0.010 V s^−1^. The current is normalised by the mean oxide surface area, which is estimated from the nanoparticle loading (250 μg cm^−2^) and the SSA shown in Table S2. LaMnO_3_ displays two characteristic responses at 0.9 V and 0.5 V, which have been ascribed to the reduction of Mn from the initial oxidation state to a 2+ state.^9^, ^10^, ^11^,^24^ The incorporation of Ba has a strong influence in the Mn redox responses for *x* higher than 0.15. Although a slight variation in the peak current ratio, there is very little difference in the voltammograms of LaMnO_3_ and La_0.85_Ba_0.15_MnO_3_. This is a somewhat surprising result given that XPS responses reveal a significant A‐site segregation in the case of La_0.85_Ba_0.15_MnO_3_ with respect to the pure lanthanide. Although the trends observed on Mn surface population estimated by electrochemical and photoemission measurements are qualitatively consistent in the case of La_x_Ca_1‐x_MnO_3_,^10^ a fully quantitative correlation remains challenging. Such a discrepancy has also been reported by Xu *et al*. who found a correlation between ORR activity and Mn surface density as determined by cyclic voltammetry but not with the XPS responses.^25^ While the Mn redox responses mainly probe the first atomic layer, photoemission responses arise from few atomic layers from the surface where significant variation in the elemental ratio may take place. On the other hand, the dampening of the Mn responses in La_0.70_Ba_0.3_MnO_3_ and BaMnO_3_ is rather significant to be exclusively rationalised in terms of surface segregation. Previous studies comparing Sr, Y and Ca manganite perovskites also show a significant influence of the A‐site in the redox Mn responses.^19^ It is clear that the surface Mn coordination is affected by the nature of the A‐site, however, quantitative assessment of the first atomic layer in these materials remains a formidable challenge.

Figure 3b illustrates the effective number density of redox active Mn sites (Γ_Mn_) as a function of the bulk Ba content, estimated from integration of the voltammetric responses as exemplified in Figure S4.^9^, ^10^, ^11^,^14^,^25^ We assume that the Mn surface sites are reduced to a Mn^2+^ state from an average initial state calculated from the bulk composition and a ABO_3_ stoichiometry. We acknowledge that the composition of the first atomic layer, including oxygen content, is expected to be difference from the bulk. However, we anticipate that the dispersion in the electrochemical measurements and in the surface composition in the ensemble of particles is larger than the error incurred in our assumption. Further details of the calculation are summarised in Table S4. To obtain Γ_Mn_, the number of Mn atoms estimated from the charge in the voltammograms is normalised by the specific surface area of the oxides calculated by TEM (Table S2). LaMnO_3_ and La_0.85_Ba_0.15_MnO_3_ exhibit comparable Γ_Mn_ values, whereas La_0.70_Ba_0.15_MnO_3_ and BaMnO_3_ show a lower value due to the increased Ba content.

**Figure 4:**
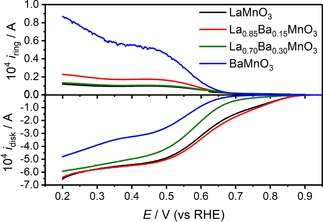
Disk (*i*_disk_) and ring (*i*_ring_) current as a function of potential of Vulcan‐supported La_*x*_Ba_1‐*x*_MnO_3_ electrodes at 1600 rpm in O_2_‐saturated 0.1 M KOH and a scan rate of 10 mV s^−1^.

Figure 4 compares the activity for O_2_ reduction in 0.1 M KOH at the different catalysts recorded with a rotation rate of 1600 rpm. LaMnO_3_ and La_0.85_Ba_0.15_MnO_3_ exhibit an ORR onset potential significantly more positive than La_0.7_Ba_0.3_MnO_3_ and BaMnO_3_. The RRDE responses recorded for the glassy carbon electrode and in the presence of the mesoporous carbon layer under identical conditions are shown in Figure S5. It is interesting to notice that the onset potential for ORR overlaps with the Mn redox signals for all the synthesised samples, as illustrated in Figure S6. This further supports the notion that the ORR to OH^−^ at these oxides is triggered by changes in the redox state of surface Mn sites. It is also observed that the diffusion limiting current is smaller in the case of BaMnO_3_ and La_0.70_Ba_0.30_MnO_3_, while a larger *i*_RING_ is observed in the range of 0.2 to 0.65 V. Using the collection coefficient of the RRDE electrode, the effective number of transferred electrons (*n*) and the hydrogen peroxide yield (% $OH_{2}^{-}$ ) were calculated and displayed in Figure S7. The trend in OH_2_^−^ yield is BaMnO_3_>La_0.70_Ba_0.30_MnO_3_>La_0.85_Ba_0.15_MnO_3_>LaMnO_3_, with *n* values close to 4 for LaMnO_3_ and La_0.85_Ba_0.15_MnO_3_ while BaMnO_3_ is close to 3 at potentials around 0.6 V. The observation that BaMnO_3_ exhibits a mixed selectivity towards both ORR mechanisms is consistent with a previous study by Yang *et al*.,^25^ who reported that BaMnO_3_ nanorods exhibited a degree of selectivity towards the 2e^−^ mechanism. It should also be mentioned that contributions from the carbon support become significant at potentials more negative than 0.65 V (see Figure S5).^9^

Figure S7 shows the Koutecky‐Levich plots for the different electrodes, which display a complex behaviour associated with the interplay between the two and four electron ORR. The slopes observed for LaMnO_3_ are closer to that for a 4e^−^ reduction. On the other hand, La_0.85_Ba_0.15_MnO_3_, La_0.70_Ba_0.30_MnO_3_ and BaMnO_3_ show a change in slope with the increase of rotation rate, implying a combination of the two reactions taking place. In order to minimise the contribution of the carbon support to the kinetic analysis describe below, we shall focus on the electrochemical responses at a potential of 0.65 V. However, we acknowledge that the contribution of the carbon support to the overall current is not entirely negligible at this potential in the case of BaMnO_3_.

**Figure 5:**
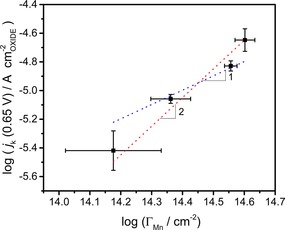
Kinetic limiting current at 0.65 V vs. RHE normalised by the mass of oxide in the electrode as a function of the effective Mn atomic surface density (*Γ*_Mn_). The slope value suggests that the rate limiting step is second order with respect to the number density of reducible Mn sites.

Figure 5 shows the kinetic limiting current density (*j*_k_) at 0.65 V normalised by the oxide real surface area as a function of Γ_Mn_. *j*_k_ is estimated by extrapolating the faradaic current to infinite angular rotation based on Koutecky‐Levich formalism (as illustrated in Figure S8). This representation clearly shows that increasing the number density of reducible Mn sites leads to an increase in the overall activity of the catalysts. Intriguingly, the slope in the logarithmic plot is significantly closer to a value of 2 than 1, suggesting that the rate limiting step in the process is second order with respect to the number density of redox active Mn sites. To the best of our knowledge, such a correlation has not been reported in the context of ORR at non‐metallic catalysts, further demonstrating the importance understanding the chemical nature of the first atomic layer the active sites at oxide nanoparticles.

Although establishing a detailed mechanistic analysis is beyond the scope of this work, it could be argued that a second‐order dependence on reducible Mn surface sites is more consistent with the so‐called “side‐on” mechanism in which O_2_ bridges to B‐sites.^6^ This mechanism is also consistent with the paramagnetic properties of LaMnO_3_ at room temperature,^26^ as well as the high tendency towards the 4e^−^ ORR pathway which is a key defining aspect to Mn based perovskites. Interestingly, DFT calculations by Ahmad et al. concluded that bridge oxygen binding is the most stable adsorption mode at fivefold coordinated Mn sites at the (101) surface.^29^ The “side‐on” mechanism is different to the “end‐on” process, not only on the fact that O_2_ binds a single B‐site, but also on the sequence of elementary steps which can lead to the formation of HO_2_^−^.^5^ Finally, our findings further reinforce the notion that electron occupancy at Mn sites during ORR is rather different to the equlibrium configuration of manganites at room temperature, thus any approach to rationalising activity of these materials from first‐principles must incorporate this important aspect..

## 3 Conclusions

The electrocatalytic activity of phase pure La_1‐*x*_Ba_*x*_MnO_3_ (0≤*x*≤1) nanoparticles towards ORR was assessed as a function of the number density of reducible Mn sites. The nanostructures were characterised by XRD, TEM, EDX and XPS, providing a thorough analysis of their crystal structure, bulk and surface composition. The formation of phase pure mixed La/Ba A‐site perovskites required higher temperatures than in LaMnO_3_ and BaMnO_3_. From XPS analysis, temperature above 900 °C promotes a significant segregation of the A‐site to the surface, with Ba segregating more extensively than La. On the other hand, cyclic voltammetry shows a gradual decrease in the effective number density of reducible Mn sites (Γ_Mn_) with increasing Ba bulk content above *x*>0.15. The ORR activity of the oxide nanoparticles supported at carbon electrodes, as probed by rotating ring‐disk electrodes, shows a significant dependence on the bulk Ba content. The larger activity was obtained for x=0 and 0.15, which is consistent with the larger Γ_Mn_ value. Interestingly, the kinetically limited current at 0.65 V shows a square dependence on Γ_Mn_ for this family of compounds. This trend suggests that the rate determining step in ORR is second order with respect to the number of redox active Mn sites. Our analysis concludes that beyond controlling particle size and surface composition on these complex materials, it is crucially important to determine and control the coordination of the Mn‐site in the first atomic layer.

## Conflict of interest

The authors declare no conflict of interest.

## Supporting information

As a service to our authors and readers, this journal provides supporting information supplied by the authors. Such materials are peer reviewed and may be re‐organized for online delivery, but are not copy‐edited or typeset. Technical support issues arising from supporting information (other than missing files) should be addressed to the authors.

Supplementary
