# Supplementary material for: Effect of Ba Content on the Activity of La1‐xBaxMnO3 Towards the Oxygen Reduction Reaction
Source: ChemElectroChem. 2018 Apr 6;5(14):1922–7. doi: 10.1002/celc.201800052 (PMC6146913; doi:10.1002/celc.201800052)
Supplement: Supplementary file 1 — Supplementary [file CELC-5-1922-s001.pdf]

Supporting Information

© Copyright Wiley-VCH Verlag GmbH & Co. KGaA, 69451 Weinheim, 2018

Effect of Ba Content on the Activity of $La_{1-x}Ba_xMnO_3$ Towards the Oxygen Reduction Reaction

Gael. P. A. Gobaille-Shaw, Veronica Celorrio,* Laura Calvillo, Louis J. Morris, Gaetano Granozzi, and David. J. Fermín*© 2018 The Authors. Published by Wiley-VCH Verlag GmbH & Co. KGaA. This is an open access article under the terms of the Creative Commons Attribution License, which permits use, distribution and reproduction in any medium, provided the original work is properly cited.An invited contribution to a Special Issue on Non-Precious-Metal Oxygen Reduction Reaction Electrocatalysis

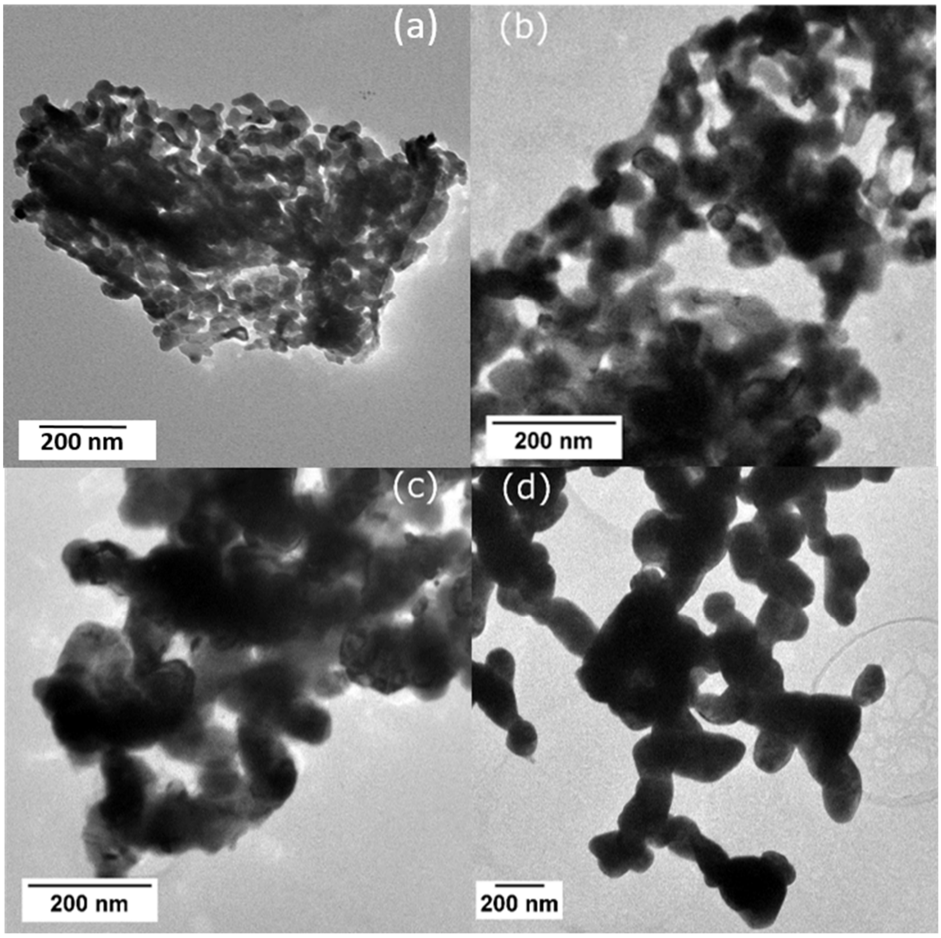

Figure S1. TEM images of $LaMnO_3$ (a), $La_{0.85}Ba_{0.15}MnO_3$ (b), $La_{0.7}Ba_{0.3}MnO_3$ (c), and $BaMnO_3$ (d).

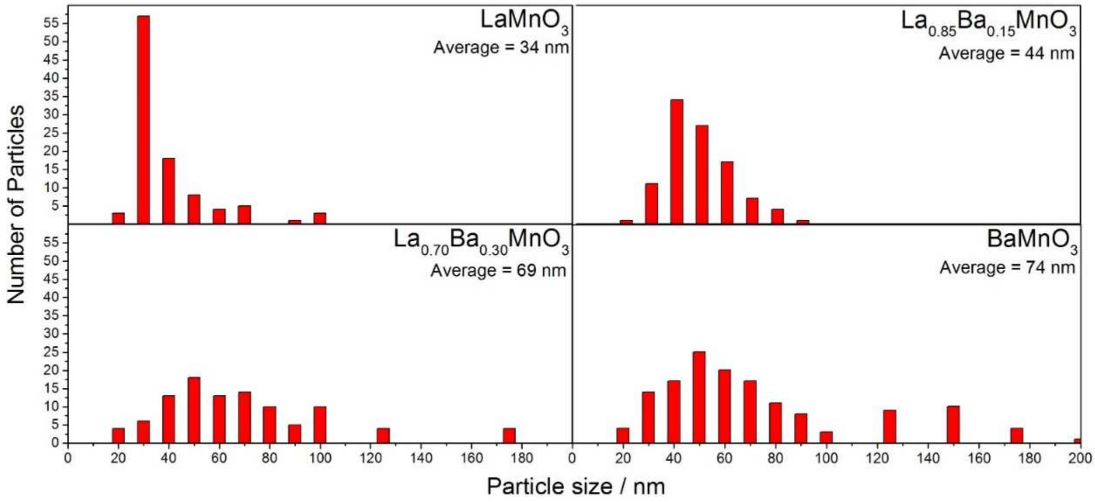

Figure S2. Average particle size and size distribution $La_{1-x}Ba_xMnO_3$ nanoparticles estimated from TEM analysis of at ~ 100 particles.

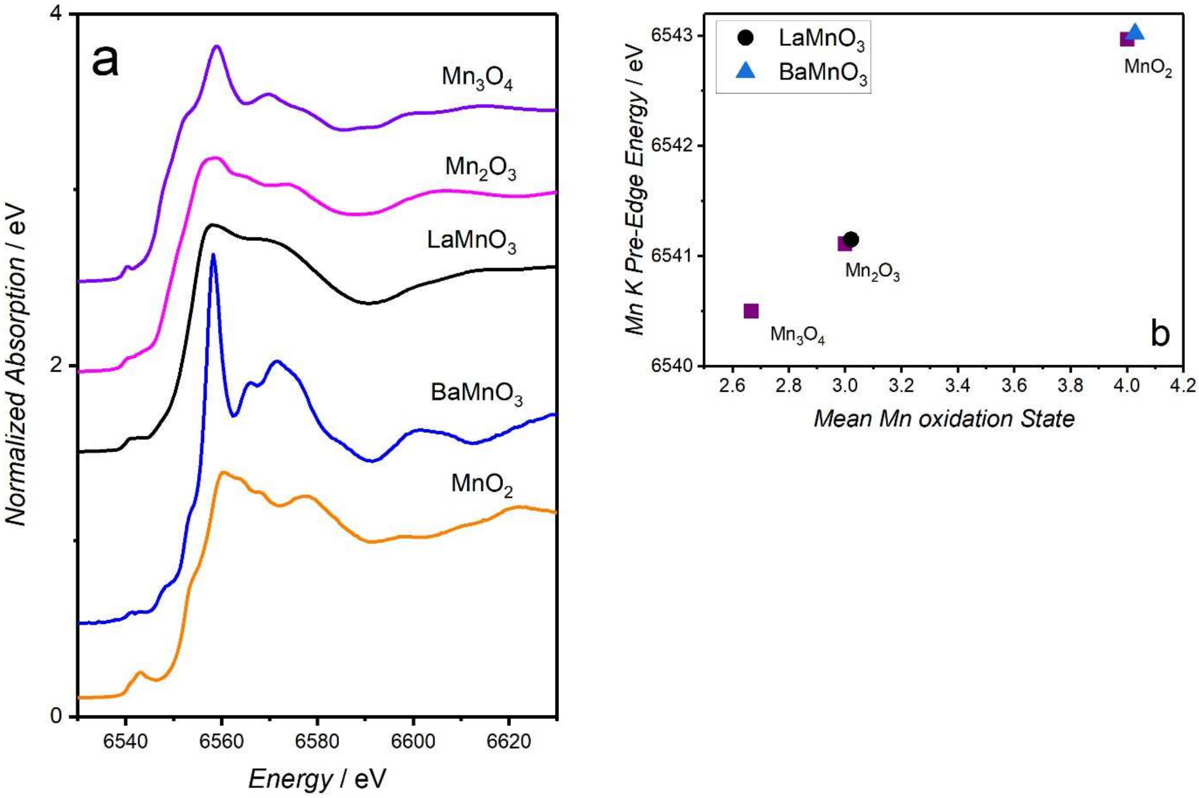

Figure S3. Normalized Mn K-edge XANES spectra of $LaMnO_3$ and $BaMnO_3$ samples together with reference manganese compounds MnO, Mn_2O_3 and MnO_2 (a). Mean Mn oxidation state as a function of the pre-edge position (b).

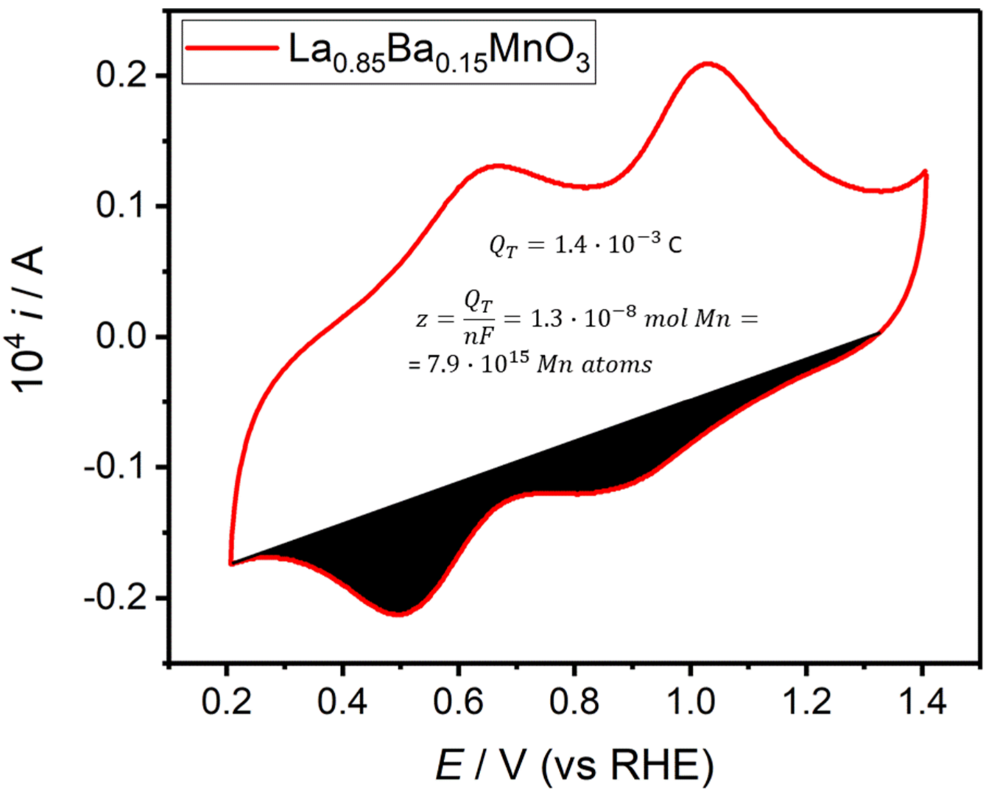

Figure S4. Calculation of the number of Mn atoms at the surface for $La_{0.85}Ba_{0.15}MnO_3$ by integration of the cathodic responses employing a linear background subtraction. The number of electrons in the reduction process (*n*) per Mn atom is given by the difference in the initial oxidation state (calculated by stoichiometry) and the final oxidation state which correspond to Mn^{2+} in all cases.

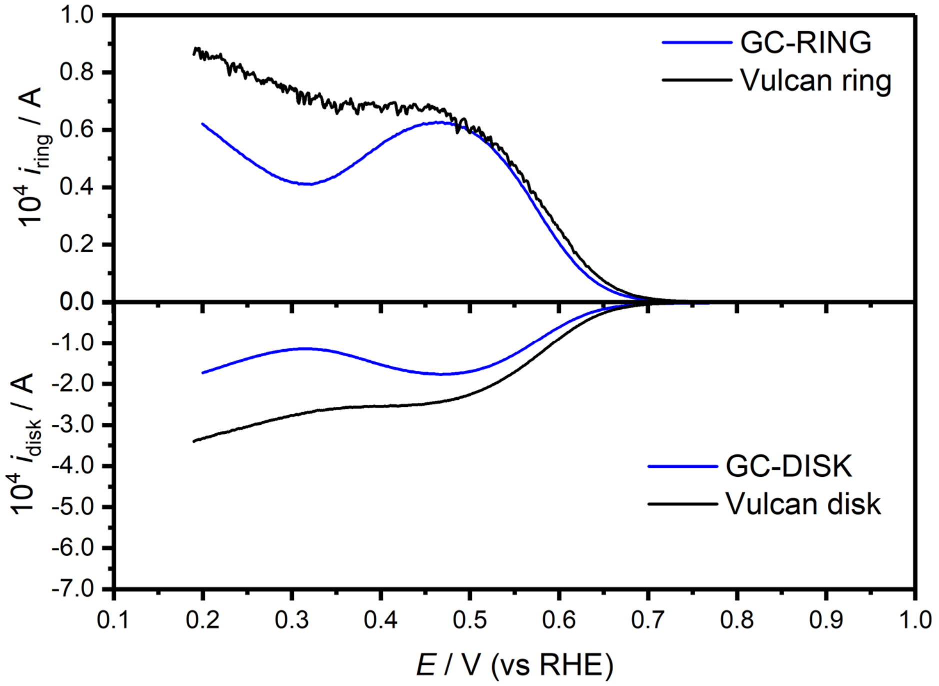

Figure S5. Rotating ring-disc curves for oxygen reduction on a Vulcan modified glassy carbon (loading 50 μg vulcan cm^{-2} and 50 μg nafion cm^{-2}) and bare glassy carbon electrodes in O_2-saturated 0.1 M KOH solution at 1600 rpm. The Pt ring was held at a potential of 1.10 V.

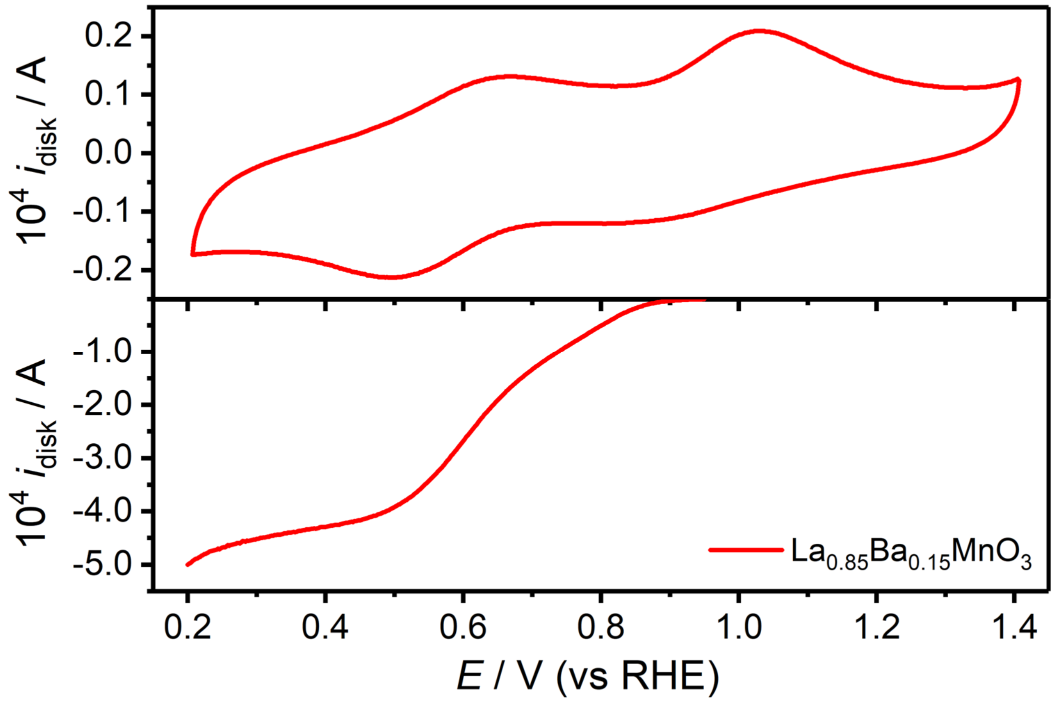

Figure S6. Cyclic voltammograms of $La_{0.85}Ba_{0.15}MnO_3$ nanoparticles at a static carbon disk electrode at 20 mV s^{-1} in Ar-saturated 0.1 M KOH (top panel), and at 1600 rpm in O_2-saturated 0.1 M KOH (bottom panel).

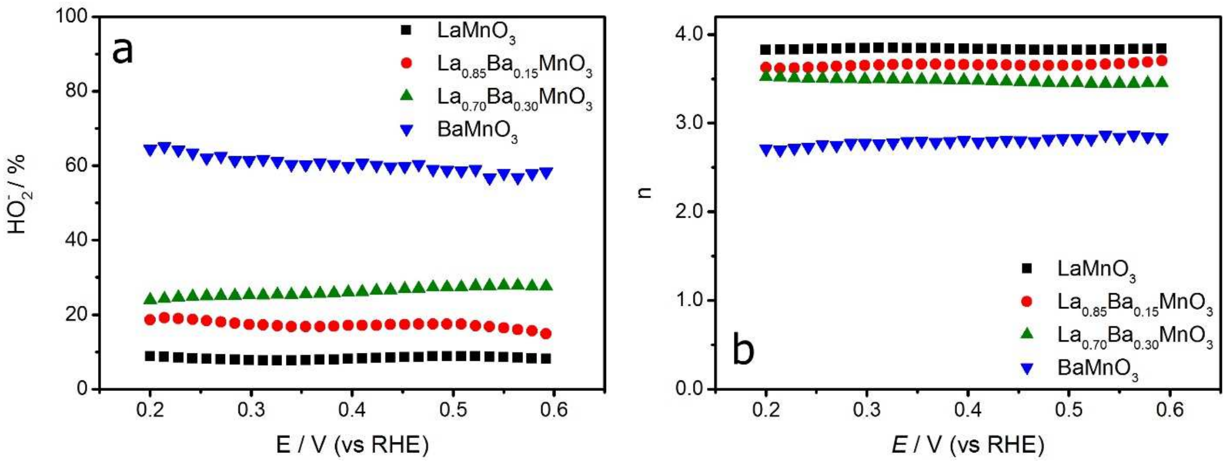

Figure S7. Peroxide yield (a) and number of electrons transferred, n (b) calculated from measurements of rotating ring-disc electrode in O_2-saturated 0.1 M KOH at 1600 rpm.

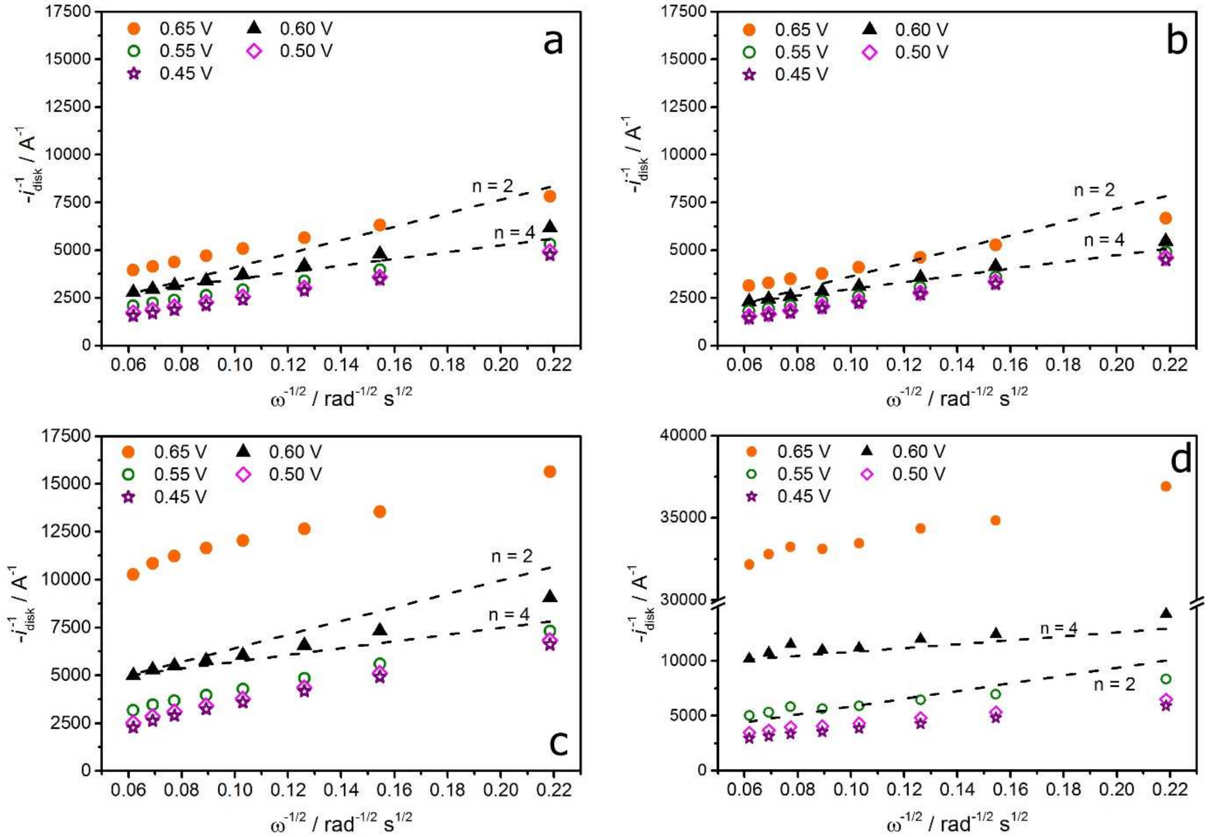

Figure S8. Koutecky-Levich plots for ORR at Vulcan supported $LaMnO_3$ (a), $La_{0.85}Ba_{0.15}MnO_3$ (b), $La_{0.7}Ba_{0.3}MnO_3$ (c) and $BaMnO_3$ (d) nanoparticles in O_2-saturated 0.1 M KOH solution at different potentials

Table S1. Bulk atomic composition of the various $La_xBa_{1-x}MnO_3$ samples as probed by Energy-dispersive X-ray spectroscopy (EDX-SEM).

Sample	Composition (% Atomic)	
	La	Ba
$LaMnO_3$	100	0
$La_{0.85}Ba_{0.15}MnO_3$	84 ± 3	16 ± 3
$La_{0.7}Ba_{0.3}MnO_3$	69 ± 2	31 ± 2
$BaMnO_3$	0	100

Table S2. Theoretical density (*ρ*) mean particle diameters (*d*) and estimated surface area (SSA) calculated assuming spherical nanoparticle geometry: SSA ($m^2\ g^{-1}$) = $6\times10^3/(\rho\times d)$

	ρ / g cm^{-3}	d / nm	SSA / $m^2\ g^{-1}$
$LaMnO_3$	6.83	33.86 ± 1.6	25.91 ± 1.3
$La_{0.85}Ba_{0.15}MnO_3$	6.81	44.4 ± 1.3	19.2 ± 0.6
$La_{0.7}Ba_{0.3}MnO_3$	6.74	69.2 ± 4.2	12.9 ± 0.8
$BaMnO_3$	5.89	73.5 ± 3.5	13.9 ± 0.7

Table S3. La:Ba:Mn atomic ratio on the surface of the $La_{1-x}Ba_xMnO_3$ samples calculated from XPS.

Sample	Composition La:Ba:Mn
$LaMnO_3$	56:0:44
$La_{0.85}Ba_{0.15}MnO_3$	56:11:33
$La_{0.70}Ba_{0.30}MnO_3$	37:28:35
$BaMnO_3$	0:49:51

From Mn3p, Ba4d and La4d with Mg K_α

Table S4. Bulk mean Mn oxidation state assuming bulk stoichiometric composition, faradaic charge associated with the reduction of surface Mn sites, and effective number of Mn atoms at the electrocatalyst surface

	Mean Mn oxidation state	Faradaic Charge / C	Mn atoms at the surface
$LaMnO_3$	+3.00	$(1.3 \pm 0.1) \cdot 10^{-3}$	$(7.9 \pm 0.3) \cdot 10^{15}$
$La_{0.85}Ba_{0.15}MnO_3$	+3.15	$(1.4 \pm 0.1) \cdot 10^{-3}$	$(7.9 \pm 0.5) \cdot 10^{15}$
$La_{0.7}Ba_{0.3}MnO_3$	+3.30	$(0.6 \pm 0.1) \cdot 10^{-3}$	$(3.0 \pm 0.4) \cdot 10^{15}$
$BaMnO_3$	+4.00	$(0.4 \pm 0.1) \cdot 10^{-3}$	$(2.0 \pm 0.7) \cdot 10^{15}$
